# Saliva—Its Characteristics in Health and Disease

**Published:** 1880-12

**Authors:** A. W. Harlan

**Affiliations:** Chicago, Ill.


### SALIVA—ITS CHARACISTICS IN HEALTH AND DISEASE.

By A. W. Harlan, D. D. S., Chicago, Ill.

A thorough knowledge of the fluids of the mouth is indispensable to the dentist if his aspirations look higher than the mere insertion of artificial dentures, or of filling teeth; as they of necessity mingle with and lubricate all our ingesta, and exert an appreciable influence over digestion, and all of them may in disease, materially interfere, with this physiological function. Moreover, none of the secretions of the alimentary tract have a higher claim upon the attention of the dentist, looked at from a pyhsiological or pathological standpoint, than the saliva; as it is believed to be the fluid which, when abnormal, assists, if it does not wholly produce caries of the teeth. Mixed saliva is a colorless, slightly clouded and somewhat viscid alkaline fluid without odor or taste. When normal it floats upon and readily mingles with water, and by this test alone we may generally expect that the mouth will be found free from inflammation, for in the latter event the saliva will at once begin to sink, until in a few moments it almost wholly deposits on the bottom of the vessel. Numerous anaylyses of saliva have been made, and of the separate fluids composing it. Parotid saliva from man contains in 1000 parts:

| | |
|---|---|
| Water | 983.308 |
| Organic matter precipitable by alcohol | 7.352 |
| Substances destructible by heat, but not precipitable by alcohol or acids | 4,810 |
| Sulpho-cyanide of potassium | 0.330 |
| Phosphate of lime | 0.240 |
| Chloride of potassium | 0.900 |
| Chloride and carbonate of sodium | 3.060 |

Submaxillary saliva is a limpid, stringy liquid, containing two parts in 100 of organic, and three parts in 100 of inorganic substances, including sulpho-cyanide of potassium. It is not a constant secretion, but is poured out in response to substances which excite the mucous membrane of the mouth or the vicinity of *Wharton's duct.* Familiar examples of its excitability are the application of the rubber dam to lower teeth, excavating a sensitive cavity, or the introduction into the mouth of sapid substance, etc. It is intimately connected with the phenomenon of gustation. Sublingual saliva is tenacious and transparent, and when cold quite gelatinous; it can hardly be called a fluid, but more nearly resembles the secretion of the mucus glands. The last two named secretions in connection with the mucus of the mouth furnish the ptyalin and mucin of mixed saliva—lastly the labial, palatal, buccal and malar glands, and membranous lining of the mouth, furnish an epithelial exudate which is true mucus and identical with that secreted by other similarly lined cavities. When the above secretions are mingled together in the mouth of a healthy man, the following materials are found in 1000 parts:

| | |
|---|---|
| Water | 994.10 |
| Solid constituents | 5.90 |
| Epithelium and mucus | 2.13 |

| | |
|---|---|
| Fat | 0.70 |
| Mucin, ptyalin and traces of alcoholic extracts | 1.41 |
| Sulpho-cyanide of potassium | 0.10 |
| Chlorides of sodium and potassium, phosphates of the alkalies and earths and oxide of iron | 2.19 |

Also small quantities of nitrogen, larger of oxygen, and still more of carbonic acid gas. Liebig thought that the saliva, on account of its containing so much oxygen was intended partially for the purpose of conveying it to the stomach. The specific gravity of saliva varies from 1,002 to 1.009, the daily quantity secreted ranges from 1.200 to 1,500 grams. Saliva normally secreted in the mouth of a healthy man, when tested at the exit of the ducts, gives an alkaline reaction, ; at times after fasting, and excessive talking, the parotid secretion, which is constant, alone is feebly acid. Alkalinity of saliva in man and animals is diminished after coitus.

Saliva is naturally an object of interest to the dentist, for he at all times and under all circumstances has to deal with it—either to mechanically exclude it during the operation of filling a tooth, or in case he finds it deficient in quality or quantity, to correct it, for he must consider it abnormal even when it parts with its inorganic salts and permits them to be deposited on the teeth in the form of calculus. The most important of the organic substances of normal saliva, is ptyalin, and of mucus, mucin. Ptyalin is the ingredient which possesses the power of converting hydrated starch into sugar; it is not coagulable by heat, but is precipitable by alcohol and is slightly soluble in water. Ptyalin instantly ceases to convert starch into sugar in the presence of an acid; it has no effect on starchy aliments that have not been boiled; it is the prime factor, in connection with mucin, of all fermentation in the mouth.

Sulpho-cyanide of potassium is the only cyanogen compound that makes its appearance physiologically in the human economy; it is an ingredient of the sublingual and submaxmillary secretions only, but Dalton states that he has found it after precipitating the solid matter of parotid saliva. Hoppe-Sayler states that sulpho-cyanic acid has been found as a decomposition product of mixed saliva. The principal interest that sulpho-cyanide of potassium possesses for us is in medico-legal inquiries. The chemist searches for meconic acid to confirm suspicion in opium poisoning, but he must first assure himself that the characteristic color is not produced by the former substance, or his results will be negative. This ingredient of saliva is slightly poisonous and not always present. A simple test for it, is to add a solution of perchloride of iron, when a beautiful red color is produced, Leucin, which is sometimes found in normal saliva, is supposed to be the mutation product of glandular structures. Its existence is denied by some chemists, but it is stated by Frey to be an occasional component of saliva In saliva, under the microscope, may be seen cast-off epithelium and gland cells with mucus and salivary corpuscles; the latter are believed to have their origin in the immediate vicinity of the tonsils, and being endowed with amœboid movement, readily mingle with the saliva. Under the influence of stimulation of the sympathetic salivary nerve filaments of the submaxillary gland, a scanty amount of viscid,alkaline secretion is expelled, containing one to two per cent. of solid cloudy matter, and very rich in mucin. Whilst irritation of the chorda-tympani branches of the nerves entering the same gland furnishes a large quantity of a non-viscid fluid and strongly alkaline in its reaction; another notable fact of the two kinds of irritation is that little colloid masses are observed as a result of the sympathetic irritation; another fact worthy of mention in this connection is that in glandular swellings the saliva is impregnated with mucilaginous and colloid matter, which must arise from the sublingual and submaxillary glands, as mucus metamorphosis is seldom, if ever, met with in the parotid glands, except as a result of external violence.

Albumen, as a constituent of saliva, may always be tested for by heat. It is in much greater quantity when the secretion is scanty; under certain circumstances the secretions of the mucus membrane of the nose and lachrymal glands mingle with the saliva and give to it various albuminous matters, yet the abundant secretion of parotidian fluid tends to keep the quantity of albumen at its usual volume; finally saliva is poured into the mouth from three pairs of glands, and numerous mucus follicles or crypts keep the membrane of the mouth and tongue moistened with an exudate which in health unites with the first named secretions to furnish a lubricant for food, which is its chief and perhaps only function until it reaches the stomach. Stepping now from the consideration of normal to abnormal secretions, it may be observed that the dentist, from the nature of his avocation, ought to be most familiar with its characteristics as such, but it is not so.

Specialists in other departments of medicine have furnished the most original and accurate observations in this field. The more one reads on the subject of abnormal secretion, the more he feels the need of fresher investigation, and more definite analyses of saliva in pathological manifestations, in spite of the fact that so much attention has already been spent upon it. It is a well-known fact that when saliva is neutral or slightly alkaline, caries of the the teeth is at a standstill, and when the reverse is the case, according to the character of the reaction, decay is progressing more or less rapidly. Simple calciferous saliva of itself does no particular harm to the teeth themselves, but the latter complications are not so to be characterized; in addition to covering a portion of the teeth with calculus, it may give rise to ranula and catarrhal inflammation of the gums, which latter disease, if allowed to care for itself, permits tartar to be deposited even on the apex of the root of the tooth, causing neuralgia; but, before the latter complication, the whole secretion is changed by becoming profuse in quantity and excessively alkaline in its reaction, denoting disturbance of nervous equilibrium.

Saliva presents the same reaction during the course of putrid fever and purpura hemorrhagica. Pending the formation of an alveolar abscess up to the climax of pain, the saliva is abundant and the reaction alkaline or neutral; but afterward it becomes acid until the subsidence of the swelling when it once more becomes normal in quantity and quality. While in general the saliva may give an acid reaction as a result of inflammation of the gums, we do not look to it, but to the hyper secretion of mucus as the cause of such reaction. The acute diseases causing hyper-secretion of mucus are acute stomatitis, amygdalitis, bronchitis, pneumonia, pharyngitis, pleurisy, acute enteritis; and those of a chronic nature are marsh fever, dyspepsia, gastralgia, dysentery, etc. The mucus secreted is not of itself so much to be feared in these affections, but by increasing the flow of saliva by the use of remedial agents, there is a larger amount of ptyalin present, which, in conjunction with the *mucus*, furnishes an active ferment, producing from azotized substances as secondary reactions, butyric, valeric, benzoic, and other acids destructive to the teeth. In simple gastric disorders saliva is generally acid, which may be accounted for by increased tissue metamorphosis, the epithelium of glandules being cast off so rapidly that a chemical change takes place in the cells, causing the secretions to appear acid.

Saliva is always scanty in fever. Thirst and suppressed secretion is a sure indication of fever in connection with heat, and is usually dependent on buccal and pharyngeal catarrh, and changes in the gastric mucus membrane. The tongue, which is dry during high fever, obstinately refuses to become moist until the temperature decreases or intestinal hemorrhage sets in, when, in spite of vascular depletion it becomes moist and remains so until increase of fever causes it to become dry again. Saliva is very copious, frothy and alkaline in apoplexy, hysteria and hydrophobia. The anomalies and abnormalities of oral secretion are numberless and instructive, but impossible to catalogue in a paper of this character. We can find parasites in it nearly always, as the thrush fungus in infancy, and in the wasting diseases—as tuberculosis, also micrococcus dentalis in whooping cough, leptothrix buccalis, vibrios and bacteria constantly. The latter stimulate the nitrogenous remains of food to putrefactive fermentation, yielding lactic acid from sugar, etc. This acid is present in saliva in gout, rheumatism, intermittent fever, diabetes and gastro-enteritis; acetic acid in scrofula, small-pox, scorbutus, apthæ and miliary fever; hydrochloric acid from immoderate use of animal flesh, including salt meats, and galvanic action, and in the early stages of inflammation of the oral mucus membrane there is an increase of the soluble chlorides which, under favorable conditions decompose, liberating the chlorine, which unites with the hydrogen present, to form the above acid. Saliva is fetid in cancrum oris and syphilitic ulcerations, and is usually acid in reaction in catarrhal inflammations and cutaneous diseases, and venereal affections; the gums in the latter are always softened and spongy, unless mercury has been exhibited. In mercurial salivation and iodism as much as two per cent. of inorganic matter with a large quantity of coagulable material have been excreted, the latter readily decomposing when lodged between the teeth and presenting an acid reaction. Uric acid has been found in saliva and occasionally urea.

In diabetes there is atrophy of the glandular organs of the mouth, throat and gums, saliva, strongly acid, and the oral membrane most likely to be the seat of scorbutus. Zenker found in a severe case of encephalitis that the mucus membrane of the tongue and throat was covered with iodium albicans. Space is wanting to further enumerate the characteristics of abormal saliva, Ammonia and sulphuretted hydrogen are nearly always present in unclean mouths. To test for the latter: add acetate of lead and there will be a black precipitate. Pregnancy, a purely physiological function, giving rise to repeated vomiting and capricious appetite, disturbs the buccal fluids to such a degree that a rapid decay of teeth is observed as a sequence. It is not to be wondered at that caries of the teeth should be so prevalent when it is remembered that no disturbance of the human system prevails for even a *short* period without changing the character of the oral secretions. The various acids, organic and inorganic, coming in contact with the teeth as aliments or remedial agents, or as the result of fermentation--which latter is favored in proportion as the saliva is scanty and the mucus abundant—renders it necessary for the dentist of to-day and his future successor to be a student of chemistry and hygiene, a preserver and a master of the laws of health.—*Trans. of the Illinois State Dental Society.*

